# Research Trends and Collaborative Patterns in *Wolbachia* and *Aedes aegypti* Studies: A Scientometric Analysis

**DOI:** 10.3390/ijerph23070862

**Published:** 2026-06-30

**Authors:** Yoon Ling Cheong, Jia Hui Lim, Mohd Hazilas Mat Hashim, Nor Syahaliyana Saidin, Shyamini Ann Samson, Mohd Khairuddin Che Ibrahim, Hui Li Lim, Farah Diana Ariffin, Han Lim Lee, Nazni Wasi Ahmad, Azahadi Omar, Kuang Hock Lim

**Affiliations:** 1 Biomedical Museum Unit, Special Resource Centre, Institute for Medical Research, National Institutes of Health, Ministry of Health Malaysia, Jalan Setia Murni U13/52, Seksyen U13 Setia Alam, Shah Alam 40170, Selangor, Malaysia; hazilas@moh.gov.my (M.H.M.H.); norsyahaliyana@moh.gov.my (N.S.S.); shyamini@moh.gov.my (S.A.S.); 2 School of Pharmacy, Faculty of Health and Medical Sciences, Taylor’s University, Subang Jaya 47500, Selangor, Malaysia; jiahui.lim@taylors.edu.my; 3 Biomedical Research, Strategic & Innovation Management Unit, Director’s Office, Institute for Medical Research, National Institutes of Health, Ministry of Health Malaysia, Jalan Setia Murni U13/52, Seksyen U13 Setia Alam, Shah Alam 40170, Selangor, Malaysia; m.khairuddin@moh.gov.my; 4 Centre of Clinical Care and Outcomes Research, Institute for Clinical Research, National Institutes of Health, Ministry of Health Malaysia, Jalan Setia Murni U13/52, Seksyen U13 Setia Alam, Shah Alam 40170, Selangor, Malaysia; lim.hl@moh.gov.my; 5 Medical Entomology Unit, Infectious Disease Research Centre, Institute for Medical Research, National Institutes of Health, Ministry of Health Malaysia, Jalan Setia Murni U13/52, Seksyen U13 Setia Alam, Shah Alam 40170, Selangor, Malaysia; farahdiana.a@moh.gov.my (F.D.A.); nazni@moh.gov.my (N.W.A.); 6 Independent Researcher, Kuala Lumpur 53100, Wilayah Persekutuan, Malaysia; mosquito7090@gmail.com; 7 Special Resource Centre, Institute for Medical Research, National Institutes of Health, Ministry of Health Malaysia, Jalan Setia Murni U13/52, Seksyen U13 Setia Alam, Shah Alam 40170, Selangor, Malaysia; drazahadi@moh.gov.my; 8 Independent Researcher, Seri Kembangan 43300, Selangor, Malaysia; keelimkota@yahoo.com

**Keywords:** *Aedes aegypti*, *Wolbachia*, vector control, scientometrics, dengue

## Abstract

**Highlights:**

**Public health relevance—How does this work relate to a public health issue?**

- The deployment of *Wolbachia* bacteria in *Aedes aegypti* serves as an alternative biological control strategy to replace or suppress wild vector populations and block arbovirus transmission to humans.
- This study uses quantitative scientometric metrics to map the global research landscape, trends, and collaborative networks, defining the explicit boundaries of empirical evidence for *Wolbachia* and *Ae. aegypti* interventions.

**Public health significance—Why is this work of significance to public health?**

- This study provides the first comprehensive scientometric mapping of the global research landscape dedicated specifically to *Wolbachia* and *Aedes aegypti*, tracking publication evolution and citation impacts from 2000 to 2025.
- This study uncovers knowledge gaps and future research priorities, allowing international health agencies to strategically optimize funding and vector control resources.

**Public health implications—What are the key implications or messages for practitioners, policy makers and/or researchers in public health?**

- This study delivers a bibliographically grounded roadmap of global research networks, key institutional hubs, and high-velocity emerging trends, serving as an evidence-based framework for future research investments and sustainable vector control policies.
- This study highlights the operational necessity of establishing national quality and biosafety standards, clear regulatory frameworks for open-field releases, and targeted sentinel systems to monitor long-term strain stability.

**Abstract:**

*Aedes aegypti* (*Ae. aegypti*) is the primary vector for dengue, Zika and chikungunya, which represent major global public health concerns. The use of *Wolbachia* as a biological control agent in *Ae. aegypti* has gained significant international attention following the successful establishment of field-released mosquitoes in Australia, Malaysia, Brazil, Indonesia and Singapore. This study presents a comprehensive scientometric analysis of the research landscape of *Wolbachia* and *Ae. aegypti*. Data comprising 662 English-language publications from 2000 to 2025 were extracted from the Scopus database. Analytic tools, including VOSviewer and R-based Biblioshiny, were employed to quantify author productivity, transcontinental collaboration networks, thematic evolution, research gaps and future directions, while Bradford’s Law of Scattering was used to identify core dissemination channels. Publications have shown a steady upward trajectory since 2000, with an overall relative growth rate of 0.3%, while annual citations peaked in 2009 and 2011 (3337 and 3460 citations, respectively). The dataset strictly conformed to Bradford’s distribution (0.16% error), identifying *PLOS Neglected Tropical Diseases* (11.9%) and *Parasites and Vectors* (5.6%) as the core journals. Global research networks are predominantly led by Australia and the United States, supported primarily by the National Institutes of Health (14.8%) and the National Health and Medical Research Council (14.2%). Crucially, thematic analysis using a methodological triangulation approach demonstrates a progressive maturation in the field, shifting from foundational laboratory mechanisms toward large-scale deployment logistics and microbiome dynamics. Overall, this study highlights the intellectual landscape, underscores the vital role of global collaboration, and provides strategic insights to guide future evidence-based policies in *Wolbachia–Aedes aegypti* research.

## 1. Introduction

Scientometrics is the discipline that applies quantitative analysis to scientific data, including publications, patents, clinical trials, grants, and other academic outputs [1]. It focuses on measuring and characterizing the quantitative features of science, scientific research and scholarly communication [2]. By applying mathematical and statistical models, scientometric evaluation assess the quality, impact, and influence of research within specialized fields [3]. Generally, such assessments help identify knowledge gaps and forecast future trends and directions within a scientific domain.

*Wolbachia* is a bacterium naturally present in over half of all arthropod species, including fruit flies, mosquitoes, nematodes, tsetse flies, bed bugs, leafcutter ants, kissing bugs, and termites [4]. The *Wolbachia* genus comprises maternally inherited endosymbionts and represents the most widespread group of intracellular bacteria in the animal kingdom [5]. It has been extensively studied in association with medically important human disease vectors such as *Aedes aegypti (Ae. aegypti)*, *Aedes albopictus (Ae. albopictus)*, *Aedes polynesiensis*, *Culex pipiens (Cx. pipiens)*, and *Anopheles stephensi*, as well as various agricultural pests [4]. It was first discovered nearly a century ago as a Rickettsia-like organism inhabiting the gonads of several insects—notably, *Cx. pipiens* mosquitoes [6]. Cowdry [7] and Hertig and Wolbach [6] have described *Wolbachia* as a Gram-negative, intracellular, Rickettsiae-like bacteria localized in the germline and somatic tissues of various insects and other arthropods [4]. The first sequenced *Wolbachia* genome was the *w*Mel strain from *Drosophila melanogaster*, belonging to supergroup A [8]. *Wolbachia* can modulate the biology of its host cells and has evolved mutualistic relationships through cytological interactions with host tissues [9]. Scientific interest in the bacterium was revived in 1956 when Laven [10] demonstrated that the reproductive incompatibility observed among certain mosquito isolates, known as cytoplasmic incompatibility (CI), was driven by this maternally inherited, antibiotic-curable organism.

The application of *Wolbachia* in pest and vector management has gained significant attention globally. Two main approaches are employed in releasing *Wolbachia*-infected *Ae. aegypti* populations: population replacement and population suppression. In the population replacement strategy, both male and female *Ae. aegypti* carrying *Wolbachia* are released to replace the wild population, ensuring that all offspring inherit the bacterium and possess reduced vector competence [11]. Conversely, population suppression involves releasing only *Wolbachia*-infected males; when they mate with wild females, the resulting eggs fail to hatch due to CI [12]. Both strategies have been successfully implemented worldwide: population replacement of *Ae. aegypti* in Australia, Brazil, Indonesia, Vietnam, and Malaysia and population suppression of the same species in Singapore and China [13,14]. Additionally, the sterile insect technique (SIT) has been proposed as a complementary approach to the incompatible insect technique (IIT) for the management of tephritid pest species using *Wolbachia* [15].

Prior bibliometric evaluations have explored related scopes but have skipped specific focus on this vector. Vittori and Dominko [16] assessed the research output and collaboration patterns in studies on terrestrial isopods, utilizing VOSviewer and CitNetExplorer to visualize networks of keywords and individual citations. Silva Salustino et al. [17] conducted a bibliometric analysis of biological control strategies using *Wolbachia* in fruit flies, covering the period 2016 to 2020. Using the Science Citation Index Expanded, Halim et al. [18] evaluated dengue vector research from 2010 to 2020, noting that publication output peaked between 2014 and 2016. Furthermore, Wu et al. [19] addressed the knowledge gap in biological control research in China, with *Wolbachia* emerging as one of the 14 major clusters within biological control studies.

Scientometric research focused on *Wolbachia* and *Ae. aegypti* is vital because *Ae. aegypti* is the primary vector of dengue, Zika, chikungunya and other arboviral diseases that pose a massive global public health burden. While *Wolbachia* has been extensively studied as a biocontrol agent, existing scientometric analyses focus on either general *Wolbachia* research [20], other insect hosts such as fruit flies [17], or unrelated invertebrates [16]. There is currently no scientometric study examining the global research landscape specifically with respect to *Wolbachia* in *Ae. aegypti*. The findings will provide insights into existing knowledge gaps, highlight emerging research priorities, and support evidence-based policy-making and vector control strategies, ensuring that efforts and resources are focused on the most impactful areas.

The general objective of this study is to conduct a rigorous scientometric analysis of the scientific literature published on *Wolbachia* and *Ae. aegypti* to uncover global trends and collaborative networks. The specific objectives include the following: (i) to analyze the trends of annual distribution of the scientific literature and citation trajectories; (ii) to identify the most prolific authors and their levels of collaboration; (iii) to assess the level of collaboration across countries and institutions; and (iv) to investigate the evolution of research keywords and thematic trends over time.

## 2. Materials and Methods

### 2.1. Data Retrieval and Screening Strategy

**Figure 1:**
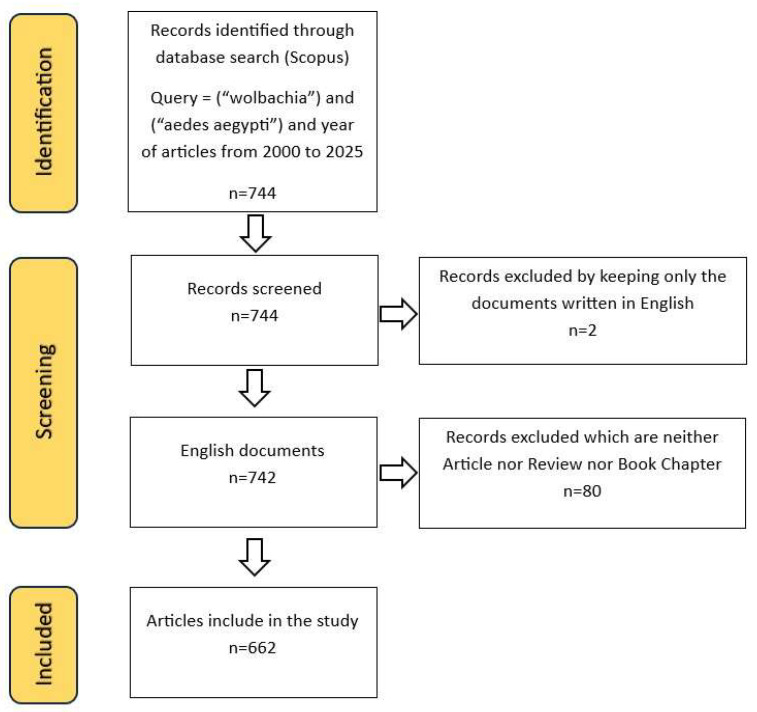
Flow diagram of the selection of articles.

Publication data were extracted from Elsevier’s Scopus database on 30 June 2025, using the search query (“*Wolbachia*”) AND (“*Aedes aegypti*”). The temporal parameters were restricted to publication years ranging from 2000 to 2025, which initially resulted in the retrieval of a total of 744 records. To refine the dataset, explicit inclusion and exclusion criteria were systematically applied during the screening phase: documents not written in English were removed (*n* = 2), and the selection was strictly limited to peer-reviewed document types—specifically, articles, reviews, books, and book chapters—resulting in the exclusion of irrelevant publication formats (*n* = 80). Following this step-by-step filtering process, a final dataset of 662 articles was obtained for subsequent analysis. This selection process is visually summarized in the flow diagram (Figure 1).

### 2.2. Relative Growth Rate

The relative growth rate (RGR) was analyzed using Microsoft Excel, measuring the change in the number of publications per year using the following formula:

$$RGR=\frac{ln\left(N_{1}\right)-ln(N_{2})}{t_{2}-t_{1}}$$

where *t*_1_ is the initial time period, *t*_2_ is the final time period, *N*_1_ denotes the cumulative number of publications at time *t*_1_, and *N*_2_ denotes the cumulative number of publications at time *t*_2_.

### 2.3. Bradford’s Law

Bradford’s Law was applied to examine journal productivity in relation to the dispersion of articles across journals. The journals were ranked in descending order of the number of articles published, then divided into three zones following the connection pattern expressed as 1:*n*:*n*^2^, where n is the Bradford multiplier. The most productive journals form the core (nucleus), and the remaining journals are allocated to subsequent zones [21].

### 2.4. Performance Analysis

Descriptive and quantitative analyses were conducted using Microsoft Excel Professional Plus 2021, VOSviewers version 1.6.20 [22], Biblioshiny via R software version 4.4.1, and Python version 3.12.7. Key scientometric information, including article counts, citation counts, journal sources, author affiliations, funding agencies, and publication details, was cleaned and verified for accuracy. A graphical dot plot of author production over time was generated using Biblioshiny. Temporal trends in topics were visualized utilizing the *ggalluvial* package in R. Furthermore, textual analysis of all abstracts was conducted to construct word clouds using Python, deploying the *wordcloud* library alongside the Natural Language Toolkit (NLTK 3.9.2).

### 2.5. Science Mapping

Co-citation analysis captured the field’s intellectual foundations, whereas keyword co-occurrence and bibliographic coupling revealed its emerging trends and thematic structure. This study adopts an analytical triangulation strategy, integrating two distinct bibliometric techniques that provide complementary views of the research landscape. First, keyword co-occurrence analysis is used to delineate the conceptual structure of the research by identifying which topics frequently appear together [23]. Second, bibliographic coupling reveals the intellectual structure of the domain by examining how individual publications are connected based on the foundational references they share [23]. Integrating these techniques enables us to cross-verify the relationships between the active research topics being published today and the underlying academic foundations that connect them.

VOSviewer software was employed to visualize the co-citation network of cited references, co-occurrence clusters of author keywords, and bibliographic coupling of documents. For the 225 articles lacking author keywords, indexed keywords from the dataset were utilized as substitutes. Common general terms (e.g., “article”, “animals”, and “human”) were replaced with blanks in the thesaurus file.

### 2.6. Topical Trend Analysis

To understand how research on *Wolbachia* and *Ae. aegypti* has shifted over time, VOSviewer was utilized to map and analyze how often different author keywords appear together in scientific papers using co-occurrences analysis. First, the raw data were cleaned using a keyword dictionary in text format to combine matching terms, fix plurals, and remove duplicates. Only keywords that appeared at least twice were included, leaving 835 unique author keywords grouped into 18 different topic areas (clusters). Each word’s importance was tracked by counting how many times it showed up (Weight <Occurrences>) and how strongly it connected to other terms in the network (Weight <Total link strength>).

Next, the timeline of these topics was tracked using the overlay visualization map saved from the co-occurrence analysis, with keywords color-coded by their average publication year (Score <Avg. pub. year>). To pinpoint the most important new trends, words that mostly appeared in 2021 or later were filtered, and their citation impact (Score <Avg. citations norm.>) was evaluated. Finally, research gaps were identified by isolating for key biological, field-testing, or geographical words that had unusually low connection scores compared to the main topics in the network.

## 3. Results

### 3.1. Publication Trends

Publications on *Wolbachia* in *Ae. aegypti* research have shown a steady upward trend since 2000, with an overall RGR of 0.3%. Total citations peaked in 2009 and 2011, reaching 3337 and 3460 citations, respectively, indicating that highly influential papers originated during these years (Figure S1). The data for 2025 cover only the first half of the year and do not represent a full-year count.

### 3.2. Author Production

The most prolific author in *Wolbachia*-*Ae. aegypti* research was Ary Hoffmann from the University of Melbourne, Australia, with 83 publications, 6073 citations, an h-index of 37, a g-index of 77 and an m-index of 2.176 (Table 1). The second most productive author was Scott O’Neill from Monash University, Australia, who published 65 articles; accumulated 10,330 citations; and achieved an h-index of 41, a g-index of 65 and an m-index of 1.577. Elizabeth A. McGraw from Pennsylvania State University recorded a rapid, high-impact contribution in recent years, matching O’Neill’s m-index despite starting later in the field (Figure S2).

### 3.3. Top Cited Papers

The most cited publication was contributed by Moreira et al. (2009) [24], titled “A *Wolbachia* symbiont in *Aedes aegypti* limits infection with Dengue, Chikungunya, and Plasmodium” in *Cell*, receiving 1311 citations (Table S1). The second most highly cited article was by Hoffmann et al. (2011) [25], “Successful establishment of *Wolbachia* in *Aedes* populations to suppress dengue transmission” in Nature, with 1155 citations. Walker et al. (2011) [26] ranked third with their *Nature* paper. The top-five most cited articles were all published between 2009 and 2011.

Focusing on the most recent decade (2015–2025), a Brazilian study demonstrating that *Wolbachia* blocks Zika virus led, with 398 citations [27], followed by an Australian study on the epidemiological efficacy of *Wolbachia* deployments [28] (Table S2). A gene-drive study by Champer et al. (2019) [29] ranked third, followed by a study by Nazni et al. (2019) [30] on the establishment of the *w*AlbB strain in Malaysia, with 254 citations, highlighting high regional research impact in Asia [30].

### 3.4. Country Collaboration and Top Institutions

**Figure 2:**
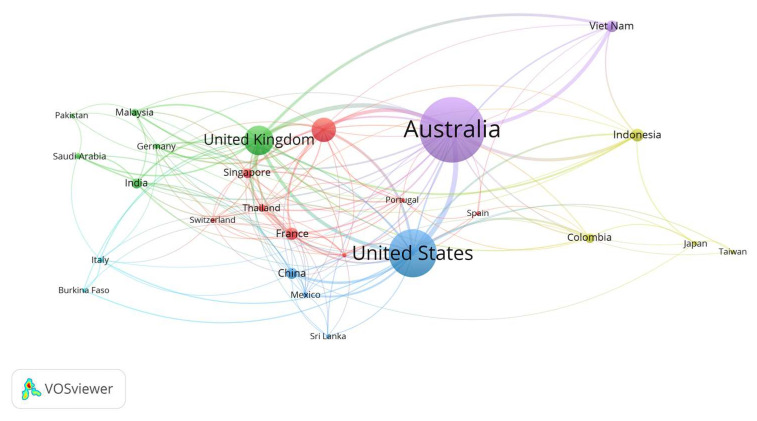
Country collaboration network based on co-authorship of *Wolbachia*-*Ae. aegypti* research from 2000 to 2025.

The country co-authorship network (Figure 2) reveals 26 highly active countries with at least five articles distributed across six strategic collaborative clusters. This landscape is dominated by three primary macro-hubs: Australia, the United States, and the United Kingdom. The networks reveal transcontinental bilateral field deployment between Australia and Viet Nam (purple cluster); cross-Pacific research matrices led by the United States alongside China, Mexico, and Sri Lanka (blue cluster); and a Euro-Asian translational pipeline headed by the United Kingdom, along with nations like India and Malaysia (green cluster). Furthermore, integrated global consortia focus on urban disease surveillance and multi-continental field trials across the red and yellow clusters, while localized Afro-European trajectories sit on the network’s periphery within the cyan cluster (Italy and Burkina Faso).

Institutional output analysis revealed that Australian universities hold the top-three positions. The University of Melbourne yielded the highest output, with 179 articles (27.0%), followed by Monash University, with 113 articles (17.1%), and the University of Queensland, with 78 articles (12.2%). Outside of Australia, Fundação Oswaldo Cruz (Brazil) was the fourth largest contributor (9.5%), while Universitas Gadjah Mada (Indonesia) emerged as the most productive Asian institution (3.9%), securing a position among the top-10 global institutes (Table 2).

### 3.5. Bradford’s Law of Scattering: Journal Distribution

**Figure 3:**
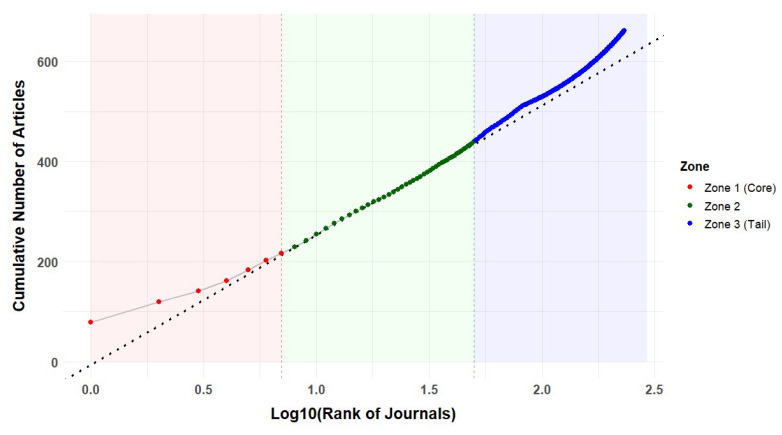
Bradford’s Law of Scattering curve for publications on *Wolbachia* in *Ae. aegypti* (2000–2025) (N = 662 articles). The journals are divided into three Bradford zones: Zone 1, representing the highly productive core journals (red); Zone 2, representing moderately productive journals (green); and Zone 3, representing marginally productive peripheral journals (blue). The black dotted line indicates the ideal logarithmic–linear trend predicted by Bradford’s Law based on Zone 2.

The 662 articles were divided into three zones. As shown in Table 3, Zone 1 (core journals) comprised 7 journals contributing 216 articles (32.6%). Zone 2 (allied journals) included 43 journals with 223 articles (33.7%). Zone 3 (peripheral journals) consisted of 183 journals accounting for 223 articles (33.7%). Figure 3 shows the Bradford’s Law cumulative curve for publications on *Wolbachia* and *Ae. aegypti* (2000–2025).

According to Bradford’s law, the distribution of journals across zones follows the theoretical proportion expressed as 1:*n*:*n*^2^. In this study, the observed distribution of journals across the three zones was found to be 7:43:183. Given a nucleus of seven journals and a calculated mean Bradford multiplier (n) of 5.20, the expected distribution ($7\;:7n\;:7n^{2}$) yields 7 :36.40 :189.23, summing to an expected total of 232.63 journals.

The mathematical precision of the model was validated by calculating the percentage of error between the observed and expected totals:

$$Percentage\;of\;Error=\left|\frac{Observed\;Total-Expected\;Total}{Observed\;Total}\right|\times 100$$

$$Percentage\;of\;Error=\frac{\left|233-232.63\right|}{233}\times 100=0.1588\%\approx 0.16\%$$

The exceptionally low error margin (0.16%) confirms that the dataset perfectly fits Bradford’s law.

### 3.6. Top Journals

*PLoS Neglected Tropical Diseases* was the most prominent outlet, publishing 79 articles (11.9%), followed by *Parasites and Vectors* (39 articles; 5.6%) and *Scientific Reports* (22 articles; 3.3%) (Table 4).

### 3.7. Top Funders

The National Institutes of Health (NIH), Maryland, USA, funded the highest number of publications (*n* = 98 articles, 14.8%), closely followed by the National Health and Medical Research Council (NHMRC), Australia (*n* = 94, 14.2%), and the US Department of Health and Human Services (HHS) (*n* = 78, 11.8%) (Table 5).

### 3.8. Wordcloud of All Abstracts

The bigram analysis (Figure S3a) indicated that “dengue virus”, “vector control”, “*aegypti* mosquitoes”, and “*Wolbachia* strain” are dominant keywords. In trigram sequences (Figure S3b), “sterile insect technique”, “cytoplasmic incompatibility CI”, “endosymbiotic bacterium *Wolbachia*”, “vector control strategies” and “dengue, chikungunya, Zika” ranked highest.

### 3.9. Keywords Evolution by Period

**Figure 4:**
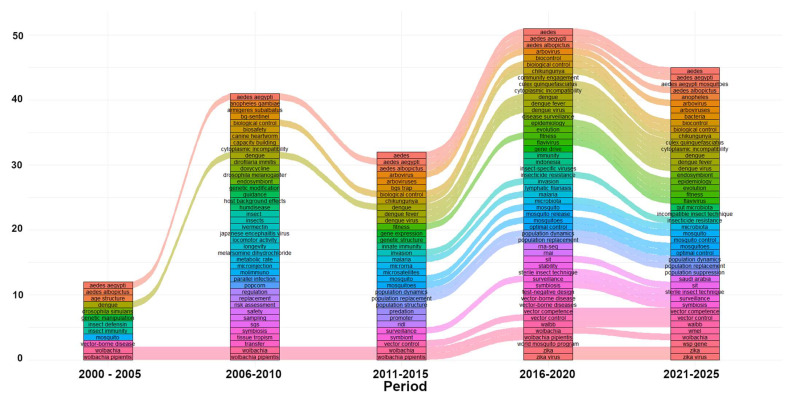
Thematic evolution of publication keywords on *Wolbachia* and *Aedes aegypti* across five periods.

The thematic evolution mapping (Figure 4) reveals structural shifts of research trends over time. From 2000 to 2005, research focused on exploratory tools and basic science, including *Drosophila simulans*, genetic manipulation, age structure, insect defensins and insect immunity. Between 2006 and 2010, terms like *Anopheles gambiae*, *Armigeres subalbatus*, BG-sentinel, canine heartworm, *Dirofilarial immitis*, doxycycline, *D. melanogaster*, endosymbiont, genetic modification, guidance, host background effects, humdisease, ivermectin, Japanese encephalitis virus, locomotor activity, longevity, melarsomine dihydrochloride, metabolic rate, microinjection, molimmuno, parallel infection, popcorn, regulation, replacement, risk assessment, safety, sampling, SGS, symbiosis and tissue tropism gained prominence. From 2011 onwards, terms transitioned sharply into applied fields, bringing *Ae. albopictus*, arbovirus, chikungunya, dengue fever, dengue virus, fitness, invasion, malaria, population dynamics, population replacement, surveillance and vector control into prominence. In the most recent period (2021–2025), the focus pivoted toward highly specialized biocontrol and genetic targets. This is evidenced by the introduction or heightened prominence of terms like *Anopheles*, *bacteria*, *endosymbiont*, *gut microbiota*, *incompatible insect techniques*, *mosquito control*, *population suppression*, *wMel*, and the *wsp gene*, alongside localized field applications in *Saudi Arabia*. Notably, CI represents a highly resilient theme across the timeline for both 2006–2010 and 2016–2025.

### 3.10. Co-Citation Analysis: Foundational Knowledge Base

**Figure 5:**
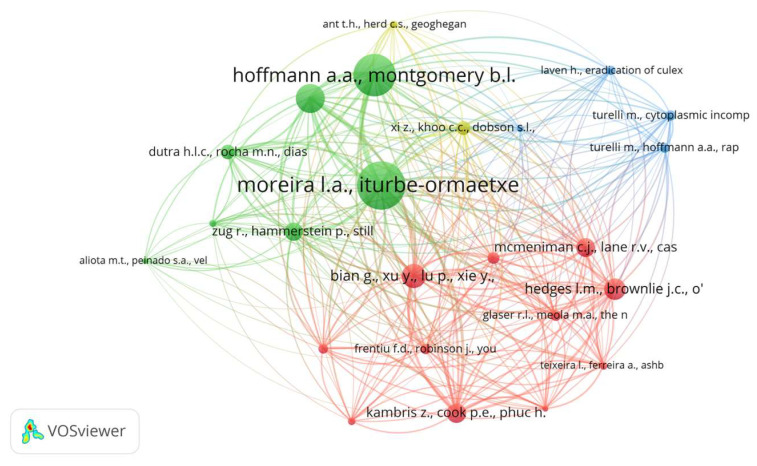
Nomological network of co-citation clusters. *Notes:* Co-citation cluster 1 (red): Biological mechanisms of viral resistance. Co-citation cluster 2 (green): Field application and transmission blocking. Co-citation cluster 3 (blue): Reproduction dynamics and cytoplasmic incompatibility. Co-citation cluster 4 (yellow): Strain evaluation and optimization.

Co-citation network analysis, with a minimum of 19 citations for a cited reference from the corpus of 662 articles, revealed four non-overlapping foundational clusters establishing the intellectual framework of the domain (Figure 5, Table 6). The red cluster (Cluster 1) on *biological mechanisms of viral resistance* is the largest, containing 11 articles focused on the *biological mechanisms of pathogen interference*, led by Bian et al. (74 citations) [31], Hedges et al. (66 citations) [32], and Kambris et al. (59 citations) [33]. The green cluster (Cluster 2) consists of seven articles centered on *field applications and transmission blocking*, containing the landmark field-trial results of Moreira et al. (148 citations) [24], Hoffmann et al. (130 citations) [25], and Walker et al. (89 citations) [26]. The blue cluster (Cluster 3), concerning *reproductive dynamics and CI models* of the *Wolbachia* strain, is led by Turelli (32 citations) [34], Turelli and Hoffmann (31 citations) [35], and Laven (28 citations) [36]. Finally, the yellow cluster (Cluster 4) stresses *strain evaluation and laboratory optimization*, with contributions by Xi et al. (43 citations) [37] and Ant et al. (24 citations) [38].

### 3.11. Keyword Co-Occurrence Analysis and Bibliographic Coupling: Knowledge Produced by the Field

Applying a methodological triangulation approach, keyword co-occurrence (uncovering conceptual hot spots) was mapped alongside bibliography coupling (uncovering structural commonalities among papers) to define meta-domains of knowledge produced by the field. The knowledge produced by *Wolbachia–Ae. aegypti* research were revealed through three clusters, encompassing 34 keywords, with a minimum of 30 occurrences, signifying the themes of “*mosquito biology*, *physiology and experimental control*”, “*arboviral disease epidemiology and human transmission*” and “*cellular symbiosis and virus replication mechanisms*” (Table 7).

**Figure 6:**
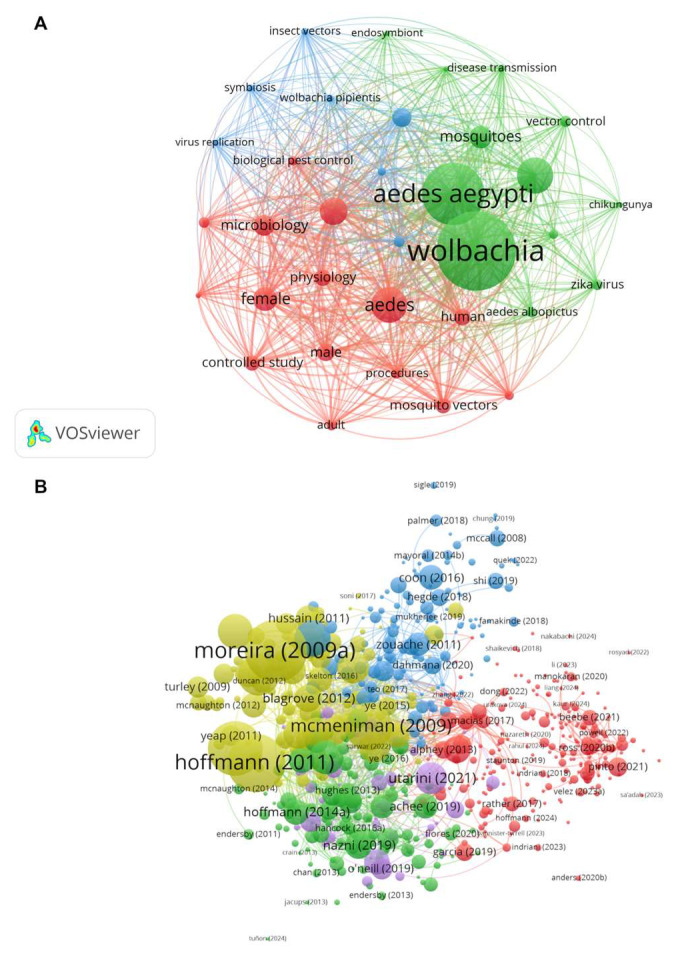
(**A**) Nomological network of keyword co-occurrence clusters. *Notes:* Co-occurrence cluster 1 (red): Epidemiology and vector control of *Aedes*-borne arboviruses. Co-occurrence cluster 2 (green): *Wolbachia*-mediated dengue suppression in *Ae. aegypti*. Co-citation cluster 3 (blue): Reproductive interventions and cytoplasmic incompatibility dynamics. (**B**) Nomological network of bibliographic coupling clusters. *Notes:* Bibliographic coupling cluster 1 (red): Large-scale mosquito introgression and mass-deployment logistics. Bibliographic coupling cluster 2 (green): Strain-specific stability and broad-spectrum virus blocking. Bibliographic coupling cluster 3 (blue): Microbiome dynamics and biological immunity mechanisms. Bibliographic coupling cluster 4 (yellow): Foundational discoveries in transinfection and pathogen interference. Bibliographic coupling cluster 5 (purple): Epidemiological efficacy and public health outcomes.

The nomological network reveals that the central themes focus on *arboviral disease epidemiology and human transmission* (keyword co-occurrence cluster 2, colored in green) (Figure 6A). On the periphery, clusters like *mosquito biology, physiology and experimental control* (keyword co-occurrence cluster 1, colored in red) and *cellular symbiosis and virus replication mechanisms* (keyword co-occurrence cluster 3, colored in blue).

#### 3.11.1. Keyword Co-Occurrence Cluster 1: Fundamental Mosquito Biology and Physiological Research

The dominant presence of “Aedes” (210 occurrences; 76.1 average citations; average publication year: 2018.3) epitomizes the foundational focus on the primary mosquito vector in laboratory and anatomical settings. The core objective of “female” (142 occurrences; 90.8 average citations; average publication year: 2017.6) and “male” (105 occurrences; 96.9 average citations; average publication year: 2017.7) underscores the comparative physiological and genetic studies crucial for understanding specific vector biology and reproduction. The emphasis on “microbiology” (125 occurrences; 71.7 average citations; average publication year: 2018.0) and “physiology” (100 occurrences; 69.9 average citations; average publication year: 2018.2) signifies the depth of scientific investigation into the internal mechanisms and native microbiota of these insects. Furthermore, the inclusion of “nonhuman” (165 occurrences; 95.8 average citations; average publication year: 2017.0) and “mosquito vectors” (93 occurrences; 34.8 average citations; average publication year: 2021.3) reflects a sustained experimental approach relying on laboratory models to inform broader scientific frameworks.

#### 3.11.2. Keyword Co-Occurrence Cluster 2: Arboviral Epidemiology and Wolbachia-Based Vector Control

The dominant presence of “Wolbachia” (469 occurrences; 49.3 average citations; average publication year: 2018.8) epitomizes the widespread scientific attention given to this endosymbiotic bacterium as a primary biological intervention strategy. The core objective of “Aedes aegypti” (368 occurrences; 56.5 average citations; average publication year: 2018.2) and “dengue fever” (213 occurrences; 63.1 average citations; average publication year: 2018.2) underscores the critical link between the major urban mosquito vector and the most prevalent disease it transmits. The emphasis on “vector control” (66 occurrences; 44.7 average citations; average publication year: 2019.6) and “mosquitoes” (134 occurrences; 63.2 average citations; average publication year: 2018.7) signifies the practical public health focus on the deployment of interventions to suppress disease transmission. Furthermore, the inclusion of “zika virus” (64 occurrences; 42.4 average citations; average publication year: 2019.6) and “arboviruses” (55 occurrences; 43.2 average citations; average publication year: 2019.9) reflects a multi-disease scope aimed at addressing a widening spectrum of emerging viral threats.

#### 3.11.3. Keyword Co-Occurrence Cluster 3: Virology, Virus Transmission, and Host–Symbiont Interactions

The dominant presence of “dengue virus” (114 occurrences; 76.6 average citations; average publication year: 2018.6) epitomizes the intense focus on the specific pathogen dynamics and cellular mechanisms of infection. The core objective of “virology” (65 occurrences; 76.2 average citations; average publication year: 2018.2) and “virus transmission” (50 occurrences; 84.1 average citations; average publication year: 2018.4) underscores the microscopic study of how pathogens replicate, survive, and spread within host vectors. The emphasis on “*Wolbachia* Pipientis” (44 occurrences; 81.0 average citations; average publication year: 2015.7) and “symbiosis” (42 occurrences; 93.8 average citations; average publication year: 2016.8) signifies the foundational exploration of the specific biological relationships utilized to block viral replication. Furthermore, the inclusion of “insect vectors” (41 occurrences; 200.8 average citations; average publication year: 2014.0) and “virus replication” (31 occurrences; 96.0 average citations; average publication year: 2016.8) reflects highly cited, fundamental research detailing the cellular bottlenecks viruses face within insects.

Using a bibliographic coupling of articles based on their similarities, the knowledge produced by *Wolbachia–Ae. aegypti* research was categorized into five clusters, encompassing 649 of the 662 articles in the corpus across the themes of “*large-scale mosquito introgression and mass deployment logistics*”, “*strain-specific stability and broad-spectrum virus blocking*”, “*microbiome dynamics and biological immunity mechanisms*”, “*foundational discoveries in transinfection and pathogen interference*” and “*epidemiological efficacy and public health outcomes*” (Table 8). The thematic assignment of the clusters from co-occurrences and bibliographic coupling analysis also follows the sensemaking approach suggested by Lim and Kumar (2024) [42].

The clusters at the center of the network are focused on themes of *large-scale mosquito introgression and mass deployment logistics* (red), *microbiome dynamics and biological immunity mechanisms* (blue) and *foundational discoveries in transinfection and pathogen interference* (yellow). The two overlapping clusters of *epidemiological efficacy and public health outcomes* (purple) and *strain-specific stability and broad-spectrum virus blocking* (green) signal that the biological robustness of the *Wolbachia strain* is intrinsically linked to its public health impact (Figure 6B).

While keyword co-occurrence analysis identifies emerging thematic hotspots and horizontal terminological connections, the nomological network of bibliographic coupling provides a deeper exploration of the intellectual foundations and thematic evolution of *Wolbachia–Ae. aegypti* research. By integrating these complementary techniques, the study employs methodological triangulation [42,52] to ensure that the identified research structures are both linguistically evident and bibliographically grounded, thereby enhancing the convergent validity of the findings [53]. The mapping of thematic clusters from keyword co-occurrence analysis with those from bibliographic coupling offers stakeholders a guide for navigating the evolving *Wolbachia-Ae. aegypti* landscape (Table 9).

These three distinct research domains represent the direct outcome of a methodological triangulation approach that integrates keyword co-occurrence analysis and bibliographic coupling to ensure that the identified thematic structures are both linguistically evident and bibliographically grounded within the global *Wolbachia*-*Ae. aegypti* landscape (Table 9).

The research domain of “*Laboratory baseline”* establishes the fundamental parameters for mosquito biology and experimental methodologies through biological characterization, diagnostic molecular procedures, and proof-of-concept transinfection studies. While early laboratory frameworks successfully demonstrate stable transmission and pathogen-blocking capabilities [37,39], scaling these interventions requires a comprehensive exploration of fitness costs and phenotypic variations across generations.

Addressing “*Applied biocontrol*, *scale-up* (*macro-level deployment*)”, the epidemiology of arboviral diseases and large-scale deployment logistics represent the macro-level applied biocontrol dimension, integrating the operational realities of high-throughput mass production [43], field population suppression, and direct epidemiological evaluations of human disease reduction [28,30,54]. Massive field trials across diverse geographic regions have established substantial evidence of protective efficacy against dengue, Zika, and chikungunya transmission. As global implementations scale up, field interventions are moving toward precision bio-deployment by leveraging real-time data streams such as localized spatial disease incidence, micro-weather variations, and wild vector population densities to dynamically manage releases of biological agent for maximized community protection and resource efficiency.

Investigating “*Internal immunity mechanics (Micro-level mechanics)*”, cellular symbiosis and microbiome mechanisms clarify how the endosymbiont interacts directly with the mosquito’s endogenous gut microbiota and native immune pathways to govern *Wolbachia’s* virus-blocking capacity [48,55]. Because the bacterium operates within a complex internal biological ecosystem rather than a cellular vacuum, understanding these tripartite host–microbiota–*Wolbachia* interactions remains vital in ensuring long-term intervention stability under environmental fluctuations. Continued mapping of how native bacterial diversity interacts with distinct *Wolbachia* strains supports the strategic selection or bioengineering of highly resilient symbiotic profiles, establishing more potent viral replication bottlenecks and stable CI mechanisms across geographically distinct vector populations.

### 3.12. Topical Trend Analysis: Research Gap and Future Direction

**Figure 7:**
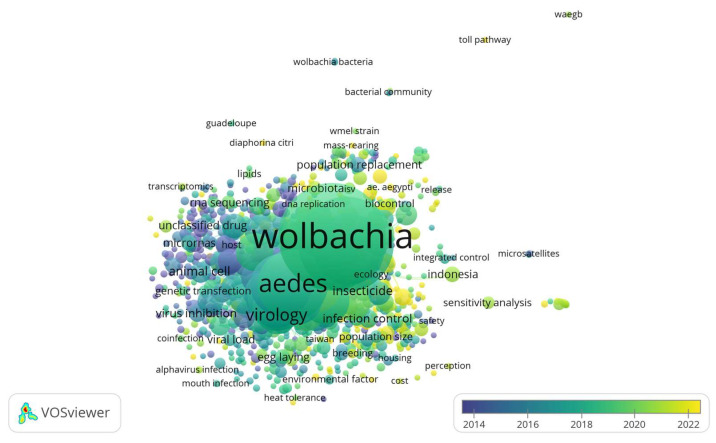
Topical trend analysis of *Wolbachia-Ae. aegypti* research.

The science mapping analysis reveals the major themes and research topics within the field, while the trend analysis delineates critical knowledge gaps across time. The topical trend analysis shown in Figure 7 reveals a clear chronological evolution from laboratory-bound basic molecular science to large-scale, real-world epidemiological application. The earliest foundation (deep blue/purple, 2014–2016) established critical cell-level mechanisms, heavily prioritizing nodes like “animal cell”, “host”, “microRNAs”, and “virus inhibition” to unpack basic pathogen-blocking dynamics. This expanded into a scaling and methodological optimization phase (teal/green, year 2018) anchored by the massive central nodes of “*Wolbachia*” and “*Aedes*”, which pivoted research toward vector ecology and deployment logistics via keywords like “biocontrol”, “population replacement”, and “mass-rearing”. The most recent frontier (yellow/light green, 2020–2022 and beyond) represents open-field execution, operational sustainability, and socio-economic integration, characterized by highly active emerging concepts like “release”, “infection control”, “safety”, “cost”, and “heat tolerance”, alongside localized deployment nodes like “Indonesia” and specialized peripheral mechanisms like the “toll pathway”.

## 4. Discussion

The findings of this scientometric research reveal significant growth and diversification in research related to *Wolbachia* and *Ae. aegypti* in recent decades. The clear growth trend reflects expanding global scientific interest, aligning closely with the World Health Organization (WHO) target product profiles (TPPs) prioritizing phase III field trials for *Wolbachia*-infected vector replacements [56].

Our institutional and country-level analyses demonstrate that intellectual architecture of this domain is highly centralized yet operationally decentralized. Australia emerged as the dominant leader in this space, anchored by the University of Melbourne, Monash University, and University of Queensland and heavily supported by national funding bodies like the NHMRC and ARC. This concentrated effort led to the seminal field verification of the *w*Mel strain’s invasion capabilities in Queensland in 2011 [25], setting a benchmark for international programs. Crucially, this geopolitical dominance is not isolated; it acts as the primary engine for global technology transfer. The country collaboration network reveals that high-resource, non-endemic macro-hubs (such as the USA, the UK, and Australia) function as analytical anchors, channeling advanced genomics, computational modeling, and core funding toward high-burden, endemic tropical nations.

This integration has underpinned major field successes, including a 69% reduction in dengue incidences in Niteroi, Brazil [44]; a 77.1% protective efficacy in Yogyakarta, Indonesia [28]; a 75.8% drop in dengue incidence across Greater Kuala Lumpur, Malaysia [57]; and a randomized controlled trial in Singapore [58]. Ultimately, these results confirm that the success of vector management policy is directly proportional to the structural density and funding stability of a nation’s international collaboration links.

This application of Bradford’s Law verified the highly concentrated nature of knowledge dissemination in this domain. The minimal deviation of 0.16% indicates strong conformity with Bradford’s theoretical distribution, suggesting that a small number of journals serve as the primary sources of knowledge dissemination in this research area. The predominant journals, *PLoS Neglected Tropical Diseases* and *Parasites and Vectors*, broadcast core findings. This structural concentration aligns tightly with established scientometric benchmarks [59,60].

The temporal evolution of keyword occurrences (Figure 4) revealed shifting research priorities over time. In the early years, studies were in an exploratory phase, investigating the potential of *Wolbachia* in mosquitoes based on earlier work on CI in *Ae. albopictus* and flies such as *Drosophila simulans* and *Drosophila melanogaster* [61,62]. The *Wolbachia* bacterium was first described in *Cx. pipiens* mosquitoes by Hertig and Wolbach [6] and later found to infect over 60% of all insect species, including *Ae. albopictus*, but notably absent in *Ae. aegypti*. Between 2006 and 2010, research attention centered on understanding the mechanism of CI and its potential to reduce host vector fecundity, with efforts focused on establishing stable *Wolbachia* infections in *Ae. aegypti* to block virus transmission. This period culminated in a major breakthrough: the successful transinfection of *Ae. aegypti* with *Wolbachia*, which paved the way for the first open-field release of *w*Mel *Ae. aegypti* in Cairns, Australia, in 2011.

Following this milestone, from 2011 onward, the targeted release of *Wolbachia*-infected mosquitoes continued to demonstrate promising effects in reducing arboviral transmission, particularly for chikungunya virus (CHIKV), which remained a key research focus [63,64]. During this period, scientists also began investigating the fitness costs associated with *Wolbachia*-infected mosquitoes, examining comparative strain performance in terms of wing length, bacterial density, insecticide resistance, starvation tolerance, and development time [65,66]. These studies were crucial in ensuring that *Wolbachia*-infected mosquitoes could compete effectively with wild-type populations and be able to survive insecticides. In the most recent decade, the research focus expanded to include the role of *Wolbachia* in blocking Zika virus transmission, particularly in response to the Zika virus epidemic of 2015–2016, which began in Brazil and spread throughout Latin America [67,68]. This period also saw increased exploration of population replacement and suppression strategies, including the SIT. Furthermore, attention has increasingly turned toward understanding the interactions between *Wolbachia* and the mosquito gut microbiota, which may influence vector competence and viral blocking mechanisms [69,70,71]. Overall, this demonstrates the progressive sophistication and diversification of *Wolbachia*-*Ae. aegypti* research.

The co-citation analysis uncovers a multi-layered body of knowledge accumulated over more than five decades, from 1967 to 2018, that forms the foundation of *Wolbachia-Ae. aegypti* research. Central to this intellectual landscape is the investigation of biological mechanisms underlying viral resistance, which illuminates how *Wolbachia* actively strengthens the mosquito’s innate defenses to block viral replication from within. This paradigm was established when Hedges et al. (2008) [32] first demonstrated that *Wolbachia* confers broad-spectrum antiviral protection in *Drosophila.* Bian et al. (2010) [31] then successfully crossed this trait into *Ae. aegypti*, demonstrating profound suppression of dengue virus replication and transmission linked directly to elevated innate immunity.

Crucially, these contemporary translational applications are deeply grounded in a long-established theoretical tradition concerning reproductive dynamics and cytoplasmic incompatibility. This historical body of literature laid the genetic groundwork for understanding how *Wolbachia* spreads sustainably through wild mosquito populations over successive generations. The historical trajectory began when Laven (1967) [36] demonstrated the practical field application of this principle by successfully eradicating a local population of filariasis vector *Culex pipiens fatigans* in Burma through the daily release of cytoplasmically incompatible males. Decades later, Turelli and Hoffmann (1991) [35] provided empirical evidence of this endosymbiont’s natural efficiency, documenting the rapid spread of a *Wolbachia* variant through California *Drosophila simulans* populations at rates exceeding 100 km per year. This ecological model was mathematically perfected by Turelli (2010) [34], who developed an analytical framework for populations with overlapping generations to calculate the “unstable equilibrium” frequency. This calculation defines the precise operational tipping point that a *Wolbachia* infection must exceed to successfully spread through, invade, and sustainably transform a wild vector population.

Building upon these foundational principles, researchers methodically compared specific *Wolbachia* strains to identify the most effective variants for blocking viral transmission while preserving host fitness. This line of empirical optimization began when Xi et al. (2005) [37] demonstrated, for the first time, that *Wolbachia* bacterial strain *w*AlbB could be successfully introduced into *Ae. aegypti*, proving that the endosymbiont could naturally drive itself through a target mosquito population. In a key development highlighting strain diversity, Ant et al. (2018) [38] identified the *w*Au variant, which exhibited superior dengue and Zika virus suppression and, crucially, maintained phenotypic stability under the high temperatures characteristic of endemic tropical regions.

These cumulative laboratory breakthroughs directly enabled the scaling of field applications and transmission-blocking strategies. Initial open-field assessments yielded vital insights. Moreira et al. (2009) [24] demonstrated that the virulent *w*MelPop-CLA strain could actively block dengue, chikungunya, and malaria parasites within the host. This suppression was linked to robust immune-system activation and potential competition for essential host-cell resources, such as intracellular cholesterol. To mitigate the severe host fitness costs associated with *w*MelPop-CLA, Walker et al. (2011) [26] resolved this trade-off by introducing the milder *w*Mel strain. This variant imposed minimal physiological costs while retaining strong CI and complete dengue blockage, allowing it to spread rapidly through semi-field cage populations. Finally, Hoffmann et al. (2011) [25] took the critical translational step of releasing *w*Mel-infected mosquitoes into natural ecosystems in Australia. The endosymbiont successfully invaded and reached near-fixation in two wild populations within months, validating its real-world capacity to reduce arboviral transmission vectors in open environments.

The analytical triangulation method highlights two principal points of discussion that carry significant implications for the future global advancement of *Wolbachia*-based vector control research. First, the mapped clusters collectively demonstrate that the field has progressively matured from early laboratory-based transinfection studies toward large-scale field deployment. This movement toward precision bio-deployment, where mass mosquito releases are guided by real-time epidemiological and entomological data, represents a promising but still evolving frontier that demands greater methodological standardization, cross-national collaboration, and adaptive implementation frameworks that are sensitive to the ecological and epidemiological diversity of dengue-endemic regions across the globe. Second, the growing scholarly attention directed toward microbiome dynamics and internal immunity mechanisms, as reflected in the bibliometric clustering patterns, points to an important re-examination of how *Wolbachia* exerts its virus-blocking effect in nature. Rather than acting in isolation, the bacterium operates within a complex biological ecosystem shaped by the mosquito’s endogenous microbiota. This understanding has far-reaching consequences for the global scientific community, as it implies that the efficacy and stability of *Wolbachia*-based interventions may vary considerably across geographically distinct mosquito populations.

The integrated meta-domains established via analytical triangulation offer direct guidance for future public health policies. The “Laboratory Baseline” highlights the necessity of rigid national quality and biosafety standardizations before biological agents leave rearing facilities. The “Applied Biocontrol and Scale-up” domain underscores the need for streamlined legal and regulatory frameworks governing open-field releases, ensuring structured community engagement, and promoting cross-border regulatory harmonization. Finally, the growing emphasis on “Internal immunity mechanics” and host–microbiome interactions underscores the critical requirement for government-funded longitudinal surveillance policies. Such mandates are essential to the monitoring of long-term strain stability in the wild and the detection of potential viral escape mutations, thereby ensuring that the intervention remains ecologically safe and epidemiologically effective across diverse global environments.

Moving forward, high-impact research must target the critical knowledge gaps revealed by our trend analysis as the field transitions from basic biological discovery to real-world epidemiological impact. These priorities can be categorized into three interconnected tiers: the fundamental research level, the applied research level and the policy and transformation research level.

At the fundamental research level, future efforts must strengthen the basic science foundation by exploring granular molecular interactions and long-term evolutionary dynamics. This requires prioritization of understudied pathways like non-coding RNAs and identification of the specific genetic loci governing virus blocking and host gene expression. This fundamental focus must also encompass the broader mosquito microbiome to understand how *Wolbachia* interacts with wild *Ae. aegypti* gut flora. Notably, while the concept of the broader “microbial community” represents a very recent frontier emerging around 2023, it remains severely under-studied in terms of absolute publication volume. Concurrently, the tracking of genomics dynamics such as wild-type introgression and the mechanisms behind unexpected infection loss is critical to ensure long-term deployment viability. Future studies should also leverage the highly active research nodes surrounding specific established bacterial variants, such as *w*Mel and *w*AlbB, to map out how distinct strains uniquely alter host phenotypes over time.

At the applied research level, investigations must bridge the gap between controlled laboratory settings and dynamic field environments by innovating scalable deployment strategies. This operational tier should increasingly focus on optimizing “egg quiescence”, “egg release”, and “optimal release programs” rather than relying solely on resource-intensive adult mosquito releases. Furthermore, the field must pursue holistic vector control by investigating “integrated pest management” and “integrated control”. The combination of *Wolbachia* deployments with traditional chemical insecticides or complementary biological agents such as *Bacillus thuringiensis* represents a vital method for sustainable suppression, as does the refined scaling of combined SIT and IIT protocols within dense urban environments.

Finally, at the policy and transformation research level, the transition of *Wolbachia* interventions from localized experimental trials to global public health standards requires that critical gaps in economics, sociology, and governance be addressed. There remains a profound need for rigorous financial justification through localized modeling, as dedicated health economics terms like “cost–benefit analysis” and “cost effectiveness analysis” are notably rare within the current literature. The establishment of sustainable global standards demands the generation of definite epidemiological proof by capitalizing on the massive citation momentum behind disease incidence and prevention data. Linking entomological field metrics to concrete human clinical outcomes such as localized declines in human illness and reductions in hospitalization rates is crucial for long-term policy adoption. Furthermore, policy frameworks must build robust systems for community engagement to address the sociological dimension of vector control. Our data reveals that “community support” represents a very recent yet severely neglected area of research. It is essential to actively break down the heavy geographic biases toward historical pioneers like Australia and Brazil to resolve regulatory, infrastructural, and socio-economic barriers faced by highly endemic, under-represented regions across South and Central America, Southeast Asia, and broader tropical zones.

This study acknowledges several limitations that should be considered when interpreting the findings. First, the scope of the analysis was deliberately confined to *Ae. aegypti* as the primary vector of interest, given the study’s core objectives. Nevertheless, the growing body of evidence supporting *Wolbachia*-mediated suppression of *Ae. albopictus* populations presents a compelling case for its inclusion in future bibliometric investigations. Second, the inclusion criteria restricted the review to peer-reviewed articles, reviews, books, and book chapters, omitting news articles, patents, gray literature, and academic awards. These excluded sources may harbor valuable insights pertaining to applied commercial innovations, policy developments, and emerging field discoveries that fall outside the current scope of this study. Third, all bibliometric data were retrieved exclusively from the Scopus database. While Scopus is widely recognized as a gold-standard citation database for peer-reviewed literature due to its comprehensive coverage, integrating records from multiple databases frequently introduces metadata inconsistencies and duplicated entries, which can severely undermine the reliability of bibliometric mapping. Fourth, this macroscopic study cannot fully capture the granular, qualitative nuances of interdisciplinary integration. Future research should prioritize narrative and systematic reviews that deeply combine biology, clinical medicine, public health, economics, and policy research to fill in the fine-grained details that a broad scientometric overview inherently misses.

Despite these limitations, this scientometric analysis offers valuable insights into the structural strengths, research gaps, and collaboration patterns shaping the *Wolbachia-Ae. aegypti* research landscape. The findings can inform evidence-based policymaking, support public health resource optimization, and foster strategic partnerships across international institutions and disciplines to advance global efforts towards sustainable, bioagent-driven vector control.

## 5. Conclusions

In conclusion, this scientometric study maps the significant growth and structural evolution of *Wolbachia* and *Aedes aegypti* research over the past two decades. The findings demonstrate a clear paradigm shift from early, laboratory-bound exploratory studies to large-scale field implementations and public health applications, though the bridging of the remaining translational gap continues to be a vital operational priority. Furthermore, our analysis highlights an increasing trend toward interdisciplinary integration, notably through the growing scholarly attention directed toward microbiome dynamics and internal immunity mechanisms. Despite inherent database and scope limitations, this study provides a bibliographically grounded roadmap of the global research landscape, key contributors, and high-velocity emerging directions, serving as a strategic guide for future research and evidence-based policy development in sustainable vector control.

## Figures and Tables

**Table 1:** Top 11 authors of papers on *Wolbachia* in *Ae. aegypti*, sorted by g-index.

Ranking	Author	Institution	No. of Publications	Citations	h-Index	g-Index	m-Index	Year Published
1	Hoffmann, Ary A.	School of Biosciences, University of Melbourne, Australia	83	6073	37	77	2.176	2009
2	O’Neill, Scott L.	Monash University, Melbourne, Australia	65	10,330	41	65	1.577	2000
3	McGraw, Elizabeth A.	Pennsylvania State University, University Park, United States	47	5478	28	47	1.556	2008
4	Simmons, Cameron P.	University College London, London, United Kingdom	46	2457	21	46	1.5	2012
5	Moreira, Luciano A.	Fundacao Oswalda Cruz, Rio de Janeiro, Brazil	41	4513	23	41	1.353	2009
6	Ritchie, Scott A.	Monash University, Melbourne, Australia	41	4410	27	41	1.8	2011
7	Asgari, Sassan	University of Queensland, Brisbane, Australia	31	1156	17	31	1.133	2011
8	Ross, Perran A.	Bio21 Institute and the Department of Genetics, University of Melbourne, Australia	31	1051	18	31	1.5	2014
9	Sinkins, Steven P.	MRC-University of Glasgow Centre of Virus Research, Glasgow, United Kingdom	25	2281	16	25	0.8	2006
10	Xi, Zhiyong	Department of Microbiology, Genetics & Immunology, East Lansing, United States	24	2240	14	24	0.667	2005
11	Anders, Katherine L.	Monash University, Melbourne, Australia	24	1342	13	24	1.625	2018

**Table 2:** Top institutional contributors to *Wolbachia*–*Ae. aegypti* research (2000–2025).

Ranking	Institution Name	Country	Number of Documents	%
1	University of Melbourne, Victoria	Australia	179	27.0
2	Monash University, Victoria	Australia	113	17.1
3	The University of Queensland, Brisbane	Australia	78	12.2
4	Fundacao Oswaldo Cruz, Rio de Janeiro	Brazil	63	9.5
5	James Cook University, Queensland	Australia	45	6.8
6	University of Oxford, Oxford	UK	28	4.2
7	QIMR Berghofer Medical Research Institute, Queensland	Australia	27	4.1
8	Australian Infectious Diseases Research Centre, Queensland	Australia	27	4.1
9	Universitas Gadjah Mada, Yogyakarta	Indonesia	26	3.9
10	Oxford University Clinical Research Unit, Ho Chi Minh City	Vietnam, UK	25	3.8

**Table 3:** Bradford’s Law distribution of publications across journal zones (2000–2025).

Zone	No. of Journals (%)	No. of Articles (%)	Bradford Multiplier
1	7 (3.0%)	216 (32.6%)	-
2	43 (18.4%)	223 (33.7%)	6.1429
3	183 (78.5%)	223 (33.7%)	4.2558
**Total**	**233 (100%)**	**662 (100%)**	**10.3987**
		**Multiplier**	**5.1993**

**Table 4:** Top journals publishing *Wolbachia–Ae. aegypti* research (2000–2025).

Rank by Articles	Journal	Number of Articles	Percentage (%)	Bradford Zone	Total Citations	Journal Impact Factor *
1	PLoS Neglected Tropical Diseases	79	11.9	Zone 1	4477	3.4
2	Parasites and Vectors	39	5.9	Zone 1	971	3.5
3	Scientific Reports	22	3.3	Zone 1	611	3.9
4	Journal of Medical Entomology	21	3.2	Zone 1	577	2
5	PLoS Pathogens	21	3.2	Zone 1	2455	4.9
6	PLoS ONE	19	2.9	Zone 1	822	2.6
7	Viruses	14	2.1	Zone 1	217	3.5
8	Frontiers In Cellular and Infection Microbiology	13	2.0	Zone 2	177	4.8
9	Insects	13	2.0	Zone 2	199	2.9
10	Proceedings of the National Academy of Sciences of the United States of America (PNAS)	13	1.7	Zone 2	1636	9.1

* Note: Journal impact factor based on wos-journal.info on 13 October 2025.

**Table 5:** Top funding agencies for *Wolbachia-Aedes aegypti* research.

Ranking	Fund Name	Country	Number of Documents	Proportion (%)
1	National Institutes of Health	USA	98	14.8
2	National Health and Medical Research Council (NHMRC)	Australia	94	14.2
3	U.S. Department of Health and Human Services (HHS)	USA	78	11.8
4	Australian Research Council (ARC)	Australia	72	10.9
5	Department of Health and Aged Care	Australia	69	10.4
6	Wellcome Trust	UK	66	9.9
7	Bill and Melinda Gates Foundation	USA	58	8.8
8	National Institute of Allergy and Infectious Diseases (NIAID)	USA	50	7.6
9	Conselho Nacional de Desenvolvimento Científico e Tecnológico (CNPq/MCTI)	Brazil	31	4.7
10	Coordenação de Aperfeiçoamento de Pessoal de Nível Superior (CAPES/MEC)	Brazil	26	3.9

**Table 6:** Foundational clusters in the *Wolbachia–Aedes aegypti* knowledge base identified via co-citation analysis.

Author(s) and Year	Article	Journal	Citations
**Co-citation cluster 1: Biological mechanisms of viral resistance**			
Bian et al. (2010) [31]	The endosymbiotic bacterium *Wolbachia* induces resistance to dengue virus in *Aedes aegypti*	PLOS Pathogens	74
Hedges et al. (2008) [32]	*Wolbachia* and virus protection in insects	Science	66
Kambris et al. (2009) [33]	Immune activation by life-shortening *Wolbachia* and reduced filarial competence in mosquitoes	Science	59
McMeniman et al. (2009) [39]	Stable introduction of a life-shortening *Wolbachia* infection into the mosquito *Aedes aegypti*	Science	58
Werren et al. (2008) [9]	*Wolbachia*: master manipulators of invertebrate biology	Nat Rev Microbiolog	37
**Co-citation cluster 2: Field application and transmission blocking**			
Moreira et al. (2009) [24]	A *Wolbachia* symbiont in *Aedes aegypti* limits infection with dengue, chikungunya, and plasmodium	Cell	148
Hoffmann et al. (2009) [25]	Successful establishment of *Wolbachia* in *Aedes* populations to suppress dengue transmission	Nature	130
Walker et al. (2011) [26]	The *w*Mel *Wolbachia* strain blocks dengue and invades caged *Aedes aegypti* populations	Nature	89
Zug and Hammerstein (2012) [40]	Still a host of hosts for *Wolbachia*: analysis of recent data suggests that 40% of terrestrial arthropod species are infected	Plos One	56
Dutra et al. (2016) [27]	*Wolbachia* blocks currently circulating zika virus isolates in Brazilian *Aedes aegypti* mosquitoes	Cell Host Microbe	45
**Co-citation cluster 3: Reproductive dynamics and cytoplasmic incompatibility**			
Turelli (2010) [34]	Cytoplasmic incompatibility in populations with overlapping generations	Evolution	32
Turelli and Hoffmann (1991) [35]	Rapid spread of an inherited incompatibility factor in *California drosophila*	Nature	31
Laven (1967) [36]	Eradication of *Culex pipiens fatigans* through cytoplasmic incompatibility	Nature	28
McMeniman and O’neill (2010) [41]	A virulent *Wolbachia* infection decreases the viability of the dengue vector *Aedes aegypti* during periods of embryonic quiescence	PLOS Negl Trop Dis	20
**Co-citation cluster 4: Strain evaluation and laboratory establishment**			
Xi et al. (2005) [37]	*Wolbachia* establishment and invasion in an *Aedes aegypti* laboratory population	Science	43
Ant et al. (2018) [38]	The *Wolbachia* strain *w*Au provides highly efficient virus transmission blocking in *Aedes aegypti*	PLOS Pathogens	24

**Table 7:** Key themes of knowledge produced by *Wolbachia–Ae. aegypti* research derived from keyword co-occurrence analysis.

Keyword	Occurrences	Average Citations	Average Publication Year
**Keyword co-occurrence cluster 1: Mosquito biology, physiology, and experimental control**			
*Aedes*	210	76.1	2018.3
nonhuman	165	95.8	2017.0
female	142	90.8	2017.6
microbiology	125	71.7	2018.0
male	105	96.9	2017.7
physiology	100	69.9	2018.2
human	98	91.3	2018.3
mosquito vectors	93	34.8	2021.3
controlled study	89	92.7	2017.3
genetics	64	46.1	2018.7
procedures	62	58.9	2019.5
adult	58	57.7	2019.1
mosquito control	56	52.4	2019.8
biological pest control	54	111.5	2017.4
polymerase chain reaction	33	80.5	2017.4
**Keyword co-occurrence cluster 2: Arboviral disease epidemiology and human transmission**			
*Wolbachia*	469	49.3	2018.8
*Aedes aegypti*	368	56.5	2018.2
dengue fever	213	63.1	2018.2
mosquitoes	134	63.2	2018.7
vector control	66	44.7	2019.6
zika virus	64	42.4	2019.6
*Aedes albopictus*	62	43.1	2018.6
arboviruses	55	43.2	2019.9
disease transmission	41	177.5	2016.0
chikungunya	31	43.5	2018.9
transmission	31	85.5	2017.0
endosymbiont	30	85.2	2017.2
**Keyword co-occurrence cluster 3: Cellular symbiosis and virus replication mechanisms**			
dengue virus	114	76.6	2018.6
virology	65	76.2	2018.2
virus transmission	50	84.1	2018.4
*Wolbachia* Pipientis	44	81.0	2015.7
symbiosis	42	93.8	2016.8
insect vectors	41	200.8	2014.0
virus replication	31	96.0	2016.8

**Table 8:** Major themes of knowledge produced by *Wolbachia–Ae. aegypti* research revealed through bibliographic coupling.

Author (Year)	Article	Source	Citations
**Bibliographic coupling cluster 1: Large-scale mosquito introgression and mass-deployment logistics**			
Crawford et al. (2020) [43]	Efficient production of male *Wolbachia*-infected *Aedes aegypti* mosquitoes enables large-scale suppression of wild populations	Nature Biotechnology	251
Pintu et al. (2021) [44]	Effectiveness of *Wolbachia*-infected mosquito deployments in reducing the incidence of dengue and other *Aedes*-borne diseases in Niterói, Brazil: A quasi-experimental study	PLoS Neglected Tropical Diseases	131
Ferguson (2018) [45]	Challenges and opportunities in controlling mosquito-borne infections	Nature	124
**Bibliographic coupling cluster 2: Strain-specific stability and broad-spectrum virus blocking**			
Dutra et al. (2016) [27]	*Wolbachia* blocks currently circulating zika virus isolates in Brazilian *Aedes egypti* mosquitoes	Cell Host and Microbe	398
Nazni et al. (2019) [30]	Establishment of *Wolbachia* Strain wAlbB in Malaysian Populations of *Aedes aegypti* for Dengue Control	Current Biology	254
Hoffmann et al. (2014) [46]	Stability of the *w*Mel *Wolbachia* Infection following Invasion into *Aedes aegypti* Populations	PLoS Neglected Tropical Diseases	232
**Bibliographic coupling cluster 3: Microbiome dynamics and biological immunity mechanisms**			
Xiaoling et al. (2011) [47]	*Wolbachia* induces reactive oxygen species (ROS)-dependent activation of the Toll pathway to control dengue virus in the mosquito *Aedes aegypti*	Proceedings of the National Academy of Sciences of the United States of America	459
Coon et al. (2016) [48]	Mosquitoes host communities of bacteria that are essential for development but vary greatly between local habitats	Molecular Ecology	222
Zouache (2011) [49]	Bacterial diversity of field-caught mosquitoes, *Aedes albopictus* and *Aedes aegypti*, from different geographic regions of Madagascar	FEMS Microbiology Ecology	190
**Bibliographic coupling cluster 4: Foundational discoveries in transinfection and pathogen interference**			
Moreira et al. (2009) [24]	A *Wolbachia* Symbiont in *Aedes aegypti* Limits Infection with Dengue, Chikungunya, and Plasmodium	Cell	1311
Hoffmann et al. (2011) [25]	Successful establishment of *Wolbachia* in *Aedes* populations to suppress dengue transmission	Nature	1155
Walker et al. (2011) [26]	The *w*Mel *Wolbachia* strain blocks dengue and invades caged *Aedes aegypti* populations	Nature	1018
**Bibliographic coupling cluster 5: Epidemiological efficacy and public health outcomes**			
Utarini et al. (2021) [28]	Efficacy of *Wolbachia*-infected mosquito deployments for the control of dengue	New England Journal of Medicine	349
O’neill et al. (2019) [50]	Scaled deployment of *Wolbachia* to protect the community from dengue and other *Aedes* transmitted arboviruses	Gates Open Research	235
Ryan et al. (2020) [51]	Establishment of *w*Mel *Wolbachia* in *Aedes aegypti* mosquitoes and reduction of local dengue transmission in Cairns and surrounding locations in northern Queensland, Australia	Gates Open Research	167

**Table 9:** Mapping of cluster findings of *Wolbachia-Ae. aegypti* research: implications for policy.

Co-occurrence (CO) Cluster	Bibliographic Coupling (BC) Cluster	Focus of Mapped Clusters	Policy Implications
**CO Cluster 1:** Mosquito biology, physiology, and experimental control	**BC Cluster 4:** Foundational discoveries in transinfection and pathogen interference	**Laboratory baseline:** Focuses on laboratory procedures like PCR and the initial proof of concept for the introduction of *Wolbachia* into *Aedes aegypti.*	**Quality and Biosafety Standardization:** Establishment of standardized national quality assurance protocols and biosafety guidelines for laboratory vector rearing, strain characterization, and pre-release fitness assessments.
**CO Cluster 2:** Arboviral diseases epidemiology and vector control interventions	**BC Cluster 1:** Large-scale mosquito introgression and mass-deployment logistics **BC Cluster 2**: Strain-specific stability and broad-spectrum virus blocking **BC Cluster 5**: Epidemiological efficacy and public health outcomes	**Applied bio-control and scale-up (macro-level application):** Centers on mass production, population suppression, and measurement of real-world reductions in human disease.	**Integration and Regulatory Frameworks:** Integration of *Wolbachia* deployments into national dengue control programs and development of legal frameworks for open-field releases, mandated community engagement, and cross-border regulatory harmonization.
**CO Cluster 3:** Cellular symbiosis and virus replication mechanisms	**BC Cluster 3:** Microbiome dynamics and biological immunity mechanisms	**Internal immunity mechanics (micro-level mechanics):** Investigation of how *Wolbachia* interacts with the mosquito’s natural microbiota and immune pathways to block virus replication and broader bacterial diversity.	**Sentinel Monitoring Systems:** Establishment of a few targeted sentinel sites for periodic sampling to monitor strain stability and efficacy, avoiding the financial burden of large-scale, mandatory ecological tracking.

## Data Availability

Data is available upon request from the author.

## Supplementary Materials

The following supporting information can be downloaded at: https://www.mdpi.com/article/10.3390/ijerph23070862/s1, Figure S1: Trends of research by total number of publication and citations for the *Wolbachia-Ae. aegypti* research from year 2000 to 2025.; Figure S2: authors’ production over time for the *Wolbachia-Ae. aegypti* research from year 2000 to 2025; Figure S3: Wordcloud of (a) bigram and (b) trigram for articles on *Wolbachia* and *Aedes aegypti;* Table S1: Top 12 most highly cited articles from 2000 to 2025 [24,25,26,27,28,31,33,37,39,47,72,73]; Table S2: Top 10 most highly cited articles from 2015 to 2025 [27,28,29,30,43,48,50,64,74,75].

## Abbreviations

The following abbreviations are used in this manuscript:

*Ae. aegypti*	*Aedes aegypti*
*Ae. albopictus*	*Aedes albopictus*
*Cx. pipiens*	*Culex pipiens*
*D. simulans*	*Drosophila simulans*
*D. melanogaster*	*Drosophila melanogaster*
CI	Cytoplasmic incompatibility
SIT	Sterile insect technique
IIT	Incompatible insect technique
RGR	Relative growth rate
NIH	National Institutes of Health
NHMRC	National Health and Medical Research Council
HHS	US Department of Health and Human Services
ARC	Australian Research Council
NIAID	National Institute of Allergy and Infectious Diseases
CNPq/MCTI	Conselho Nacional de Desenvolvimento Científico e Tecnológico
CAPES/MEC	Coordenação de Aperfeiçoamento de Pessoal de Nível Superior
WHO	World Health Organization
TPP	Target product profile
UK	United Kingdom
USA	United States of America
