# Supplementary material for: Research Trends and Collaborative Patterns in Wolbachia and Aedes aegypti Studies: A Scientometric Analysis
Source: Int J Environ Res Public Health. 2026 Jun 30;23(7):862. doi: 10.3390/ijerph23070862 (PMC13412030; doi:10.3390/ijerph23070862)
Supplement: Supplementary file 1 [file ijerph-23-00862-s001.zip › ijerph-4220549-supplementary.pdf]

Supplementary Materials

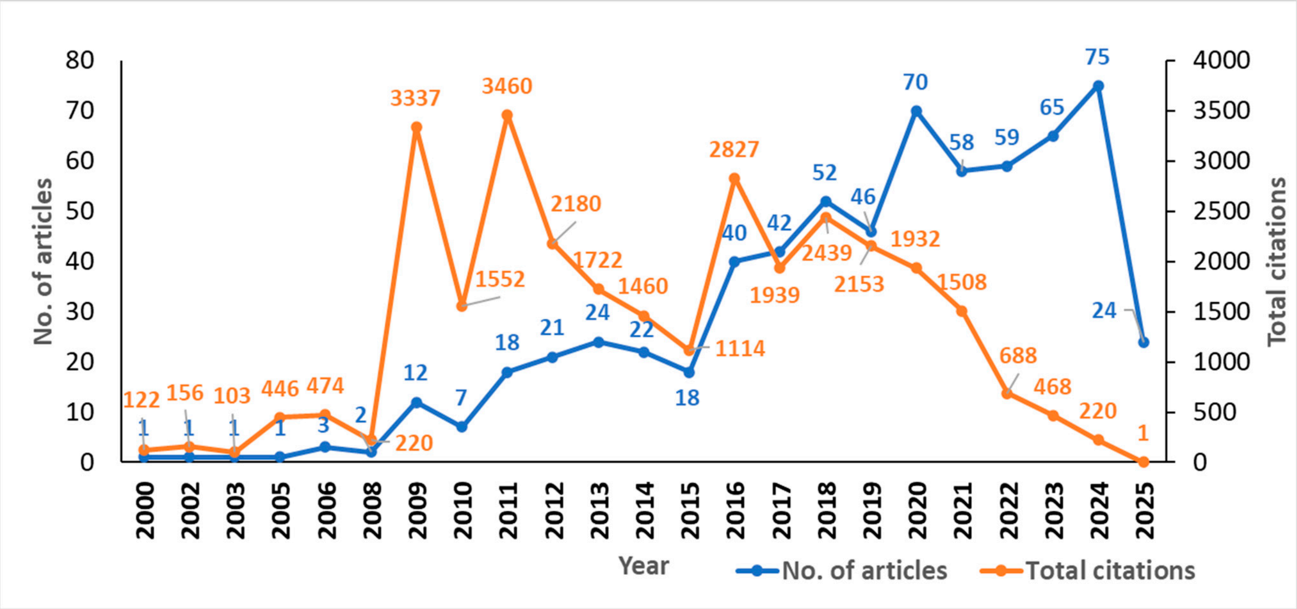

Figure S1. Trends of research by total number of publication and citations for the *Wolbachia-Ae. aegypti* research from year 2000 to 2025.

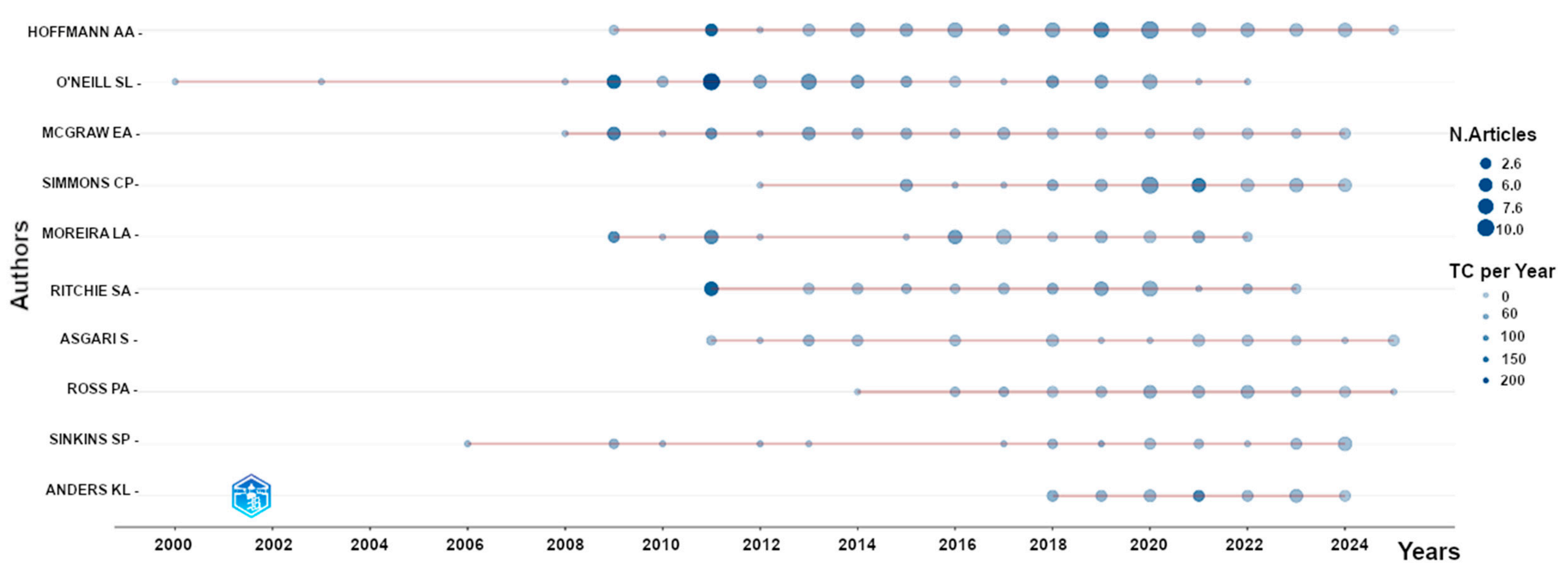

Figure S2. Authors' production over time for the *Wolbachia-Ae. aegypti* research from year 2000 to 2025.

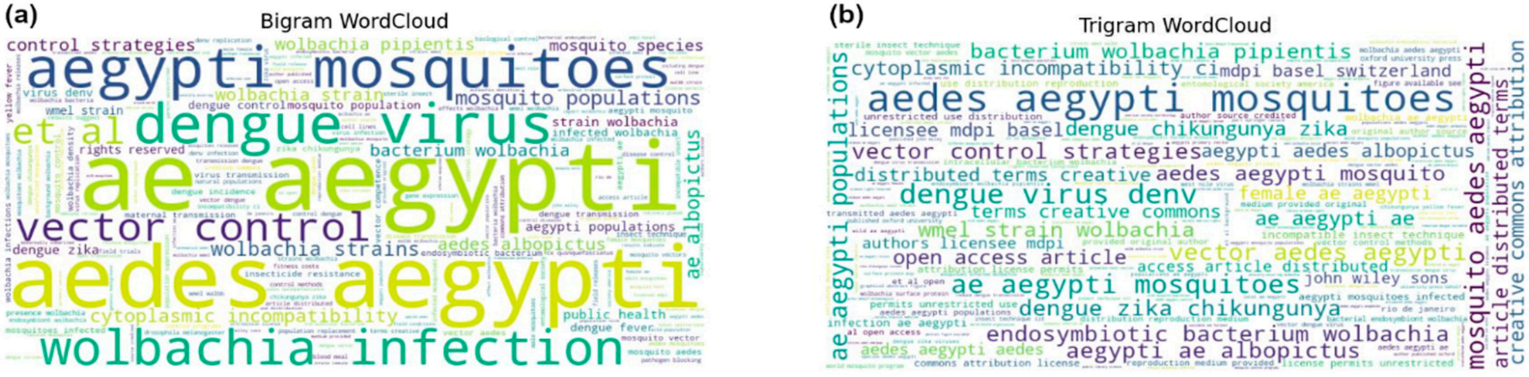

Figure S3. Wordcloud of (a) bigram and (b) trigram for articles on *Wolbachia* and *Aedes aegypti*.

Table S1. Top 12 most highly cited articles from 2000 to 2025.

	Authors	Title	Year	Total Citations (Citations per year)	Source title	Document Type
1	Moreira et al. [24]	A *Wolbachia* symbiont in *Aedes aegypti* limits infection with Dengue, Chikungunya, and Plasmodium	2009	1311 (81.93)	Cell	Article
2	Hoffmann et al. [25]	Successful establishment of *Wolbachia* in *Aedes* populations to suppress dengue transmission	2011	1155 (82.50)	Nature	Article
3	Walker et al. [26]	The *w*Mel *Wolbachia* strain blocks dengue and invades caged *Aedes aegypti* populations	2011	1018 (72.14)	Nature	Article
4	McMeniman et al. [39]	Stable introduction of a life-shortening *Wolbachia* infection into the mosquito *Aedes aegypti*	2009	767 (47.94)	Science	Article
5	Bian et al. [31]	The endosymbiotic bacterium *Wolbachia* induces resistance to dengue virus in *Aedes aegypti*	2010	603 (40.2)	PLoS Pathogens	Article
6	Xiaoling et al. [47]	*Wolbachia* induces reactive oxygen species (ROS)-dependent activation of the toll pathway to control dengue virus in the mosquito *Aedes aegypti*	2012	459 (35.31)	Proceedings of the National Academy of Sciences of the United States of America	Article
7	Xi et al. [37]	Ecology: *Wolbachia* establishment and invasion in an *Aedes aegypti* laboratory population	2005	446 (22.30)	Science	Article
8	Kambris et al. [33]	Immune activation by life-shortening *Wolbachia* and reduced filarial competence in mosquitoes	2009	417 (26.06)	Science	Article
9	Dutra et al. [27]	*Wolbachia* Blocks Currently Circulating Zika Virus Isolates in Brazilian *Aedes aegypti* Mosquitoes	2016	398 (44.22)	Cell Host and Microbe	Article
10	Utarini et al. [28]	Efficacy of *Wolbachia*-infected mosquito deployments for the control of dengue	2021	349 (87.25)	New England Journal of Medicine	Article
11	Sinkins & Gould [72]	Gene drive systems for insect disease vectors	2006	349 (31.73)	Nature Reviews Genetics	Review
12	Iturbe-Ormaetxe et al. [73]	*Wolbachia* and the biological control of mosquito-borne disease	2011	337 (24.07)	EMBO Reports	Review

Table S2. Top 10 most highly cited articles from 2015 to 2025.

	Authors	Title	Year	Total Citations (Citations per year)	Source title	Document Type
1	Dutra et al. [27]	*Wolbachia* Blocks Currently Circulating Zika Virus Isolates in Brazilian *Aedes* aegypti Mosquitoes	2016	398 (44.22)	Cell Host and Microbe	Article
2	Utarini et al. [28]	Efficacy of *Wolbachia*-infected mosquito deployments for the control of dengue	2021	349 (87.25)	New England Journal of Medicine	Article
3	Champer et al. [29]	Cheating evolution: Engineering gene drives to manipulate the fate of wild populations	2016	325 (94)	Nature Reviews Genetics	Review
4	Nazni et al. [30]	Establishment of *Wolbachia* Strain *w*AlbB in Malaysian Populations of *Aedes aegypti* for Dengue Control	2019	254 (42.33)	Current Biology	Article
5	Crawford et al. [43]	Efficient production of male *Wolbachia*-infected *Aedes aegypti* mosquitoes enables large-scale suppression of wild populations	2020	251 (50.20)	Nature Biotechnology	Article
6	O'Neill et al. [50]	Scaled deployment of *Wolbachia* to protect the community from dengue and other aedes transmitted arboviruses	2019	235 (33.57)	Gates Open Research	Article
7	Aliota et al. [64]	The *w*Mel strain of *Wolbachia* Reduces Transmission of Zika virus by *Aedes aegypti*	2016	232 (25.77)	Scientific Reports	Article
8	Achee et al. [74]	Alternative strategies for mosquito-borne arbovirus control	2019	222 (37.00)	PLoS Neglected Tropical Diseases	Review
9	Flores & O'Neill [75]	Controlling vector-borne diseases by releasing modified mosquitoes	2018	222 (31.71)	Nature Reviews Microbiology	Review
10	Coon et al. [48]	Mosquitoes host communities of bacteria that are essential for development but vary greatly between local habitats	2016	222 (24.67)	Molecular Ecology	Article
